# Functional self-assembled neocartilage as part of a biphasic osteochondral construct

**DOI:** 10.1371/journal.pone.0195261

**Published:** 2018-04-10

**Authors:** Wendy E. Brown, Daniel J. Huey, Jerry C. Hu, Kyriacos A. Athanasiou

**Affiliations:** 1 Department of Biomedical Engineering, University of California Irvine, Irvine, California, United States of America; 2 Department of Biomedical Engineering, University of California Davis, Davis, California, United States of America; University of Pittsburgh, UNITED STATES

## Abstract

Bone-to-bone integration can be obtained by osteoconductive ceramics such as hydroxyapatite (HAp) and beta-tricalcium phosphate (β-TCP), but cartilage-to-cartilage integration is notoriously difficult. Many cartilage repair therapies, including microfracture and mosaicplasty, capitalize on the reparative aspects of subchondral bone due to its resident population of stem cells and vascularity. A strategy of incorporating tissue engineered neocartilage into a ceramic to form an osteochondral construct may serve as a suitable alternative to achieve cartilage graft fixation. The use of a tissue engineered osteochondral construct to repair cartilage defects may also benefit from the ceramic’s proximity to underlying bone and abundant supply of progenitor cells and nutrients. The objective of the first study was to compare HAp and β-TCP ceramics, two widely used ceramics in bone regeneration, in terms of their ability to influence neocartilage interdigitation at an engineered osteochondral interface. Additional assays quantified ceramic pore size, porosity, and compressive strength. The compressive strength of HAp was six times higher than that of β-TCP due to differences in porosity and pore size, and HAp was thus carried forward in the second study as the composition with which to engineer an osteochondral construct. Importantly, it was shown that incorporation of the HAp ceramic in conjunction with the self-assembling process resulted in functionally viable neocartilage. For example, only collagen/dry weight and ultimate tensile strength of the chondral control constructs remained significantly greater than the neocartilage cut off the osteochondral constructs. By demonstrating that the functional properties of engineered neocartilage are not negatively affected by the inclusion of an HAp ceramic in culture, neocartilage engineering strategies may be directly applied to the formation of an osteochondral construct.

## Introduction

Over half of the 600,000 procedures performed in the United States each year to repair knee cartilage are repeat procedures, bringing to light the impact of cartilage injuries and the need for more effective therapies [1]. Unfortunately, the characteristics that give articular cartilage its mechanical functionality also contribute to its inability to heal and integrate with repair tissues [2]. A dense and integrated network of collagen and glycosaminoglycans (GAGs) provides the basis for cartilage’s mechanical properties, however, it also inhibits cellular migration. Negatively charged GAGs which impart cartilage’s ability to withstand compressive stresses approaching 10 MPa [3] also have anti-adhesive characteristics which inhibit cell adhesion and migration to the site of injury [4]. Articular cartilage is avascular; while this provides a degree of immunoprivilage which allows for allogeneic repair strategies [5], it also means that articular cartilage does not have abundant access to nutrients or progenitor cells [6]. Thus, repair relies solely on chondrocytes which have low metabolic activity, prohibiting them from mounting a sufficient healing response [7]. Additionally, the mechanically demanding environment in which articular cartilage exists may cause degeneration of repair tissue that is mechanically inferior to healthy native cartilage [8, 9]. Beyond biological factors influencing cartilage repair, failures have been attributed to insufficient graft fixation and integration [10]. Lasting repair of full-thickness cartilage defects has been reported when graft cartilage was attached to the subchondral bone and bone integrity and architecture were restored [1]. These outcomes underscore the importance of biomimetic cartilage graft fixation and integration into the surrounding native tissue, including subchondral bone. Successful articular cartilage repair may not only rely on cartilage-to-cartilage integration, but also cartilage-to-bone integration and overall graft fixation within the defect.

In contrast to cartilage, bone heals readily; many cartilage repair therapies use osteochondral approaches to capitalize on the reparative aspects of subchondral bone. Bone is vascular and therefore has access to nutrients and stem cells which are more mobile and metabolically active than chondrocytes. Additionally, bone is highly cellular and naturally undergoes constant turnover, allowing non-critical sized defects to be quickly repaired. Microfracture and mosaicplasty are among the most common cartilage repair treatments. Microfracture involves penetration of the subchondral bone to induce bleeding, allowing the cartilage defect to gain access to stem cells and other reparative factors present in the bone marrow. However, while this method results in defect filling, repair tissue is fibrocartilaginous in nature and lacks the mechanical integrity of healthy articular cartilage. With mosaicplasty, osteochondral plugs harvested from non-load bearing regions are implanted into a cartilage defect, using the bony aspect as an anchor for the graft. This technique has improved outcomes over microfracture, however it results in morbidity and fibrocartilage formation at the donor site. While osteochondral repair techniques hold promise due to their involvement of subchondral bone, they are still limited by mechanical inferiority of repair tissue and donor site morbidity.

The use of a tissue engineered, osteochondral graft may address the obstacles to cartilage repair. Ideally, an osteochondral graft should readily integrate with native subchondral bone, be mechanically stable, and allow early postoperative function under physiological loading conditions [11]. The requirements for bone healing are well studied. The optimal pore size for bone tissue ingrowth is 150–500 μm [12]. As scaffolds for osteogenesis should mimic native tissue, further engineering criteria may be gleaned from the properties of native bone, such as porosity of 50–90% and minimum compressive strength of 0.1 MPa [13, 14]. Hydroxyapatite (HAp), the main mineral constituent of bone which is commonly used as a biocompatible bone substitute [15, 16], or Beta-tricalcium phosphate (β-TCP), which is not naturally present in bone but is also used extensively as a bone substitute, may be used to engineer a ceramic with characteristics amenable to bone ingrowth, as well as provide an anchor for fixation and a nexus of regeneration for an osteochondral graft. In addition to HAp and β-TCP ceramics having customizable porosity, the resorption characteristics of a ceramic may also be tailored to match the rate of native bone remodeling to ensure long-term mechanical stability by mixing different ratios of HAp and β-TCP [17]. In addition to the ceramic phase of an osteochondral construct providing mechanical integrity to the graft upon implantation, the characteristics of scaffold-free neocartilage as the chondral phase may allow the graft to bear loading sooner after surgery than other repair methods. Neocartilage engineered with native-like structure [18], biochemical composition [19], and mechanics [20] has been reported. The use of lysyl oxidase like 2 (LOXL2) to prime neocartilage during *in vitro* culture may also be used to further address obstacles to integration by promoting collagen crosslinking across the neocartilage-native cartilage interface. A biphasic osteochondral approach including scaffold-free neocartilage as the chondral phase and an osteoconductive ceramic as the osseous phase may achieve graft fixation and stability within a cartilage defect, postoperative loading, and functional cartilage repair. However, questions remain regarding the suitability of engineering an osteochondral graft in conjunction with self-assembled neocartilage.

Given the reparative potential of tissue engineered osteochondral constructs, the objectives of this study were two-fold: The first objective was to select one ceramic composition based on comparing the ability of HAp and β-TCP ceramics formed using a novel foam casting method to promote osteochondral interdigitation and to satisfy bone ingrowth criteria. The second objective was to show that incorporation of the selected ceramic in conjunction with the self-assembling process would result in functionally viable neocartilage that would interdigitate with the ceramic. In Study 1, it was hypothesized that the β-TCP ceramic would be mechanically weaker than the HAp ceramic, but both would allow for neocartilage interdigitation. In Study 2, it was hypothesized that the use of the ceramic identified in Study 1 as the osseous phase would not alter the biochemical and mechanical functional properties of the chondral phase neocartilage.

## Materials and methods

### Ceramic fabrication

To make HAp ceramics, 22.5 g ultrafine HAp microparticles (2 μm diameter, Himed) were combined with 27 mL 10% (wt/v) Brij (Sigma) in ultrapure water and 4 mL polyethylenimine (Sigma) and vortexed overnight to form a foam. The foam was thoroughly mixed with 1 mL glycerol diglycidyl ether (Sigma) and scooped into rectangular silicon molds. This foam-containing mold was sealed in a plastic bag and put in a 40° C oven overnight to allow the blocks to solidify. Promptly after 18 hours, the block was gently removed from the mold, sealed in another plastic bag, and frozen for at least 2 hours. After the block was fully frozen, it was lyophilized for 2 days. Ball mill (1/8 inch) tool paths were developed in computer aided machining (CAM) software (Esprit). After lyophilization, the stabilized block was milled using a Computer Numerical Control (CNC) mill to 6.5 mm diameter cylindrical posts. The cylinders were then sliced to 2.5 mm thick discs using a razor blade and custom 3D-printed jig. The ceramics discs were then sintered in an oven (Nabertherm P330), which caused them to shrink approximately 24%, resulting in a diameter of 5 mm. The oven increased the temperature 1° C/minute until the temperature reached 600° C which was held for 1 hour to burn off the epoxy within the ceramic. The temperature was then increased to 1310° C at 4° C/minute and held for 8 hours before returning to room temperature (25° C).

β-TCP ceramics were made in a similar fashion, except by adding 22.5 g of ultrafine β-TCP microparticles (2 μm diameter, Himed) to 20.25 mL 10% Brij and using a slightly modified sintering scheme. In the modified sintering scheme, the oven increased the temperature 1° C/minute until the temperature reached 600° C which was held for 1 hour to burn off the epoxy within the ceramic. The temperature was then increased to 1250° C at 4° C/minute and held for 1 hour. The temperature was then decreased to 900° C at 1° C/minute and held for 24 hours before returning to room temperature (25° C).

### Ceramic characterization

The criteria required to allow for native bone ingrowth, namely, porosity of 50–90%, pore diameter of 150–500 μm, and minimum compressive strength of 0.1 MPa, served as engineering criteria for the fabricated ceramics. The porosity of each fabricated ceramic type was measured using Archimedes’ Method. Briefly, dry ceramics were weighed, and their dimensions measured. Each ceramic was then submerged in 100% ethanol under a vacuum (610 mmHg) to ensure filling of all the void spaces. After 3 minutes under the vacuum, ceramics were taken out of the ethanol, quickly blotted to remove excess fluid, and immediately weighed. Bulk porosity was calculated by Eq 1. The measured porosity of each ceramic type was then compared to the engineering criteria to allow for native bone ingrowth.

$$Bulk\;Porosity\;(\%)=\frac{\left(\frac{ceramic\;wet\;weight–dry\;weight}{density\;of\;wetting\;fluid}\right)}{bulk\;ceramic\;volume}*100$$ (1)

The diameters of the macropores within each ceramic were measured with ImageJ (National Institutes of Health) from high magnification pictures taken with a scanning electron microscope (Phenom, Nanoscience Instruments). The average diameters for each ceramic type were compared to the engineering criteria to allow for native bone ingrowth. To determine the compressive strength of each type of fabricated ceramic construct, compressive force was measure by compressing the discs between large, flat platens at a rate of 0.1 mm/minute until catastrophic, brittle failure. The experimental data was normalized by sample cross-sectional area, and a stress-strain curve was generated. The compressive strengths of the ceramics were obtained from the measured maximum stress. The evaluation of each parameter against the engineering criteria served as a method to eliminate or validate a ceramic composition to carry forward to Study 2.

### Chondrocyte isolation

Juvenile ovine articular chondrocytes (joACs) were isolated from the patellofemoral surfaces of 1-year-old Rambouillet Suffolk cross sheep obtained from a local abattoir (Superior Farms, Dixon, CA) within 48 hours of slaughter (n = 8). Cartilage from the surface of both condyles and the trochlear groove was minced into approximately 1 mm^3^ pieces and washed three times with Dulbecco’s Modified Eagle Medium containing 4.5 g/L glucose and GlutaMAX (DMEM; Gibco) and 2% (v/v) pencillin/streptomycin/fungizone (PSF; Lonza). The cartilage was then digested in 0.2% (w/v) collagenase type II (Worthington) in DMEM containing 3% (v/v) fetal bovine serum (FBS; Atlanta Biologicals) for 18 hours at 37° C with gentle rocking. After digestion, the resultant cell solutions were filtered through 70 μm cell strainers. These primary (P0) joACs were then frozen in DMEM with 20% (v/v) DMSO (Sigma) and 10% (v/v) FBS.

### Chondrocyte expansion and redifferentiation

Previously frozen P0 joACs were seeded in T-225 flasks at 1.5x10^4^ cells/cm^2^ and expanded in chemically defined chondrogenic medium (CHG medium) (DMEM containing 1% PSF, 1% ITS+ premix (BD Biosciences), 1% non-essential amino acids (Gibco), 100 nM dexamethasone (Sigma), 50 mg/mL ascorbate-2-phosphaste (Sigma), 40 g/mL L-proline (Sigma), and 100 mg/mL sodium pyruvate (Sigma) with 2% FBS and TFP supplementation (1 ng/mL TGF-β1, 5 ng/mL bFGF, 10 ng/mL PDGF; all from PeproTech) [21]. Media was exchanged every 2–3 days. At confluence, cells were lifted with 0.5% Trypsin-EDTA (Gibco) for 5 minutes followed by digestion of the cell layers with DMEM containing 0.2% collagenase type II and 2% FBS for approximately 1 hour at 37° C, triturating every 20 minutes. The resulting cell solution was filtered through a 70 μm cell strainer and reseeded into T-225 flasks at 1.5x10^4^ cells/cm^2^ to achieve three passages (P3). Passaged joACs underwent aggregate redifferentiation (P3R) as previously described [22]. Briefly, 100 mm x 20 mm petri dishes were coated with 1% (w/v) molecular biology grade agarose (Thermo Fisher Scientific) in phosphate buffered saline (PBS; Sigma) to create a non-adherent environment. Passaged joACs (750,000 cells/mL) were cultured in CHG medium containing TGB supplementation (10 ng/mL TGF-β1, 100 ng/mL GDF-5, 100 ng/mL BMP-2; all from PeproTech) in the coated petri dishes on an orbital shaker at 60 rpm for the first 3 days and remained static for the remainder of the 14-day redifferentiation period. Media was exchanged every 2–3 days. At the end of the culture period, aggregates were digested with 0.5% Trypsin-EDTA for 20 minutes, followed by 0.2% collagenase in DMEM with 2% FBS for approximately 2 hours at 37° C, triturating every 20 minutes. Following dissociation of the aggregates, cells were filtered through a 70 μm cell strainer and counted.

### Neocartilage construct seeding and culture

P3R joACs were self-assembled in non-adherent wells to form neocartilage constructs. To create the non-adherent wells, a sterile stainless-steel mold consisting of 5 mm diameter cylindrical posts was inserted into a 48-well plate, each well containing 1 mL molten 2% (w/v) agarose to create a single agarose well per plate well. After solidification of the agarose at room temperature, the mold was removed. Agarose wells were filled with CHG medium exchanged twice over the course of 5 days to ensure saturation of the agarose before cell seeding. Chondrocytes at a density of 2 million cells per 100 μL CHG medium containing 2 μM cytochalasin D (Enzo Life Sciences) and 200 units/mL hyaluronidase (Sigma) were seeded into 5 mm agarose wells. Cytochalasin D was added to the medium for the first 48 hours of culture. Constructs were unconfined on day 7 and placed into larger, agarose-coated wells. On day 10, half of the neocartilage constructs were assembled into osteochondral constructs (n = 7) and the other half (chondral control constructs; n = 7) remained in standard culture. Both chondral control and osteochondral constructs underwent treatment with 2 units/mL chondroitinase ABC (c-ABC, Sigma) for 4 hours on day 14 and lysyl oxidase-like 2 (LOXL2, Signal Chem; 0.15 μg/mL), copper sulfate (Sigma; 1.6 μg/mL), and hydroxylysine (Sigma; 0.146 μg/mL) on days 20–42. For all constructs, medium was exchanged daily prior to neocartilage unconfinement and every other day after for the rest of the duration of the 6-week culture period.

### Osteochondral construct assembly

**Fig 1:**
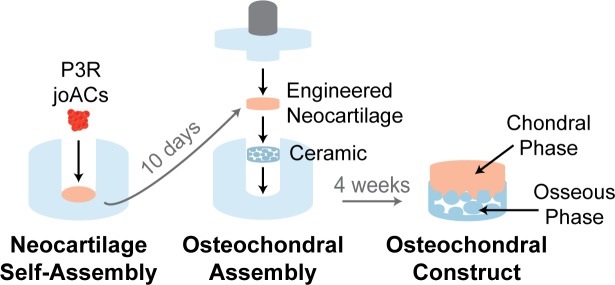
Osteochondral construct assembly. Self-assembled neocartilage constructs were placed on ceramics at day 10 after neocartilage seeding to form osteochondral constructs.

On day 10, unconfined neocartilage constructs were assembled into osteochondral constructs (Fig 1). Osteochondral assembly wells were made by injecting 4% (w/v) agarose into custom, 3D-printed molds to obtain a cylindrical, bottom agarose well and an agarose-coated cap which included a custom-made, 1 g porous, stainless-steel post. The wells and caps were placed in an excess of chondrogenic medium which was exchanged twice over 5 days to ensure saturation of the agarose. Hydroxyapatite ceramics were autoclave sterilized and pre-treated by placing them in CHG medium containing 10% FBS under a vacuum for the first 5 minutes of the 18-hour incubation to ensure penetration of the medium into the pores of the ceramic. The ceramics were washed with CHG immediately prior to osteochondral construct assembly. To assemble the osteochondral constructs, the ceramics were individually placed in the bottom of the wells, and one unconfined neocartilage construct was placed on top of each ceramic. The agarose-coated, 1 g, porous stainless-steel caps were then placed on the osteochondral assemblies and 5 osteochondral wells were placed into each well of a 6 well plate in 6 mL of CHG medium. Osteochondral constructs were cultured in these wells for an additional 14 days and were then unconfined.

### Construct gross morphological analysis

Pictures were taken of the chondral control constructs, osteochondral constructs, and cut off neocartilage after its removal from the ceramics. ImageJ was used to measure construct diameters and thicknesses from pictures. Wet weights were obtained by weighing whole chondral control constructs before samples were portioned for histological, biochemical, and mechanical analysis.

### Construct histological evaluation

Chondral control and cut off neocartilage samples were fixed in 10% neutral buffered formalin, embedded in paraffin, and sectioned along the short axis into 5 μm sections to expose the full thickness of the construct. Sections were stained with hematoxylin and eosin (H&E) to illustrate morphology, safranin O/fast green to show glycosaminoglycans (GAGs), and picrosirius red to visualize collagen. Immunohistochemistry was also performed to stain for collagen I (anti-collagen I antibody, polyclonal, rabbit, Abcam, ab34710, 1:250 dilution) and II (anti-collagen II antibody, polyclonal, rabbit, Abcam ab34712, 1:4000 dilution). Native sheep subchondral bone (rich in collagen I and void of collagen II) and articular cartilage (rich in collagen II and void of collagen I) were used as immunohistochemical control tissues. Anti-rabbit IgG secondary antibodies with an ABC-HRP peroxidase kit (Vector Laboratories) and DAB peroxidase (HRP) detection (Vector Laboratories) were used. Full thickness osteochondral samples were also fixed in 10% neutral buffered formalin, serially dehydrated in ethanol, and embedded in Technovit 7200 resin (Exakt). Sections were cut from the block and polished to 40 μm and stained with Toluidine Blue to show the neocartilage-ceramic osteochondral interface.

### Neocartilage biochemical evaluation

Chondral control and cut off neocartilage samples for biochemical evaluation were weighted to measure wet weights, lyophilized, and weighed again to measure dry weights. Dried samples were then digested with 125 μg/mL papain (Sigma), 5 mM N-acetyl-L-cysteine (Sigma), and 5 mM EDTA (Acros Organics) in phosphate buffer, pH 6.5 overnight at 60° C. Glycosaminoglycan (GAG) content was measured by a Blyscan assay kit (Biocolor). Collagen content was measured by a modified colorimetric chloramine-T hydroxyproline assay using hydrochloric acid [23]. Sircol collagen standard (Bicolor) was used to generate a standard curve. PicoGreen dsDNA reagent (Invitrogen) was used to measure DNA content. Collagen and GAG contents were normalized to wet weight, dry weight, and DNA content.

### Construct mechanical evaluation

Creep indentation compressive testing was conducted on whole osteochondral constructs and punches (3 mm in diameter) of chondral control and cut off neocartilage by applying a flat, porous indenter tip (0.8 mm diameter) using a 0.7 g load, yielding the small sample strains required for accurate data analysis. A semi-analytical, semi-numeric, linear biphasic model and finite element analysis were used to obtain the aggregate modulus, shear modulus, and permeability from the experimental data [24]. Tensile testing on chondral control and cut off neocartilage was conducted in accordance with ASTM standards (ASTM D3039). Samples were punched into dog-bone shaped specimens with gauge lengths of 1.92 mm, and paper tabs glued to the tissue outside the gauge length. The paper tabs were gripped in a TestResources machine (TestResources Inc.) and pulled at 1% of the gauge length per second until sample failure. A stress-strain curve was generated from the experimental data and the sample cross-sectional area measured via ImageJ analysis. A least-squares fit of the linear region of the curve was used to obtain the tensile modulus, and the maximum stress yielded the ultimate tensile strength (UTS). Lap shear testing was performed to measure the mechanical strength of the neocartilage-ceramic interface within the osteochondral constructs. The ceramic side of the osteochondral sample was glued to a wooden popsicle stick to provide a rigid anchor. A paper tab was glued to the neocartilage side of the osteochondral sample. The popsicle stick and paper tab were loaded into offset grips (to eliminate the creation of a bending moment) in TestResources tensile testing machine such that the neocartilage-ceramic interface was parallel to the direction of strain. The paper tab was pulled at a rate of 1 mm/min until sample failure. Sample surface areas were calculated from images of the samples and used to generate stress-strain curves. The apparent interfacial shear modulus of the osteochondral sample was obtained from the linear region of the stress-strain curve and the ultimate interfacial shear strength of the osteochondral sample was obtained from the maximum stress reached.

### Statistical analysis

All quantitative results were analyzed with a Student’s t-test (p < 0.05) in Prism 7 (GraphPad Software). A sample size of 6 per group was used. Data are presented as means with errors bars representing standard deviations. Groups not marked by the same symbol are statistically different.

## Results

### Ceramic characterization

**Fig 2:**
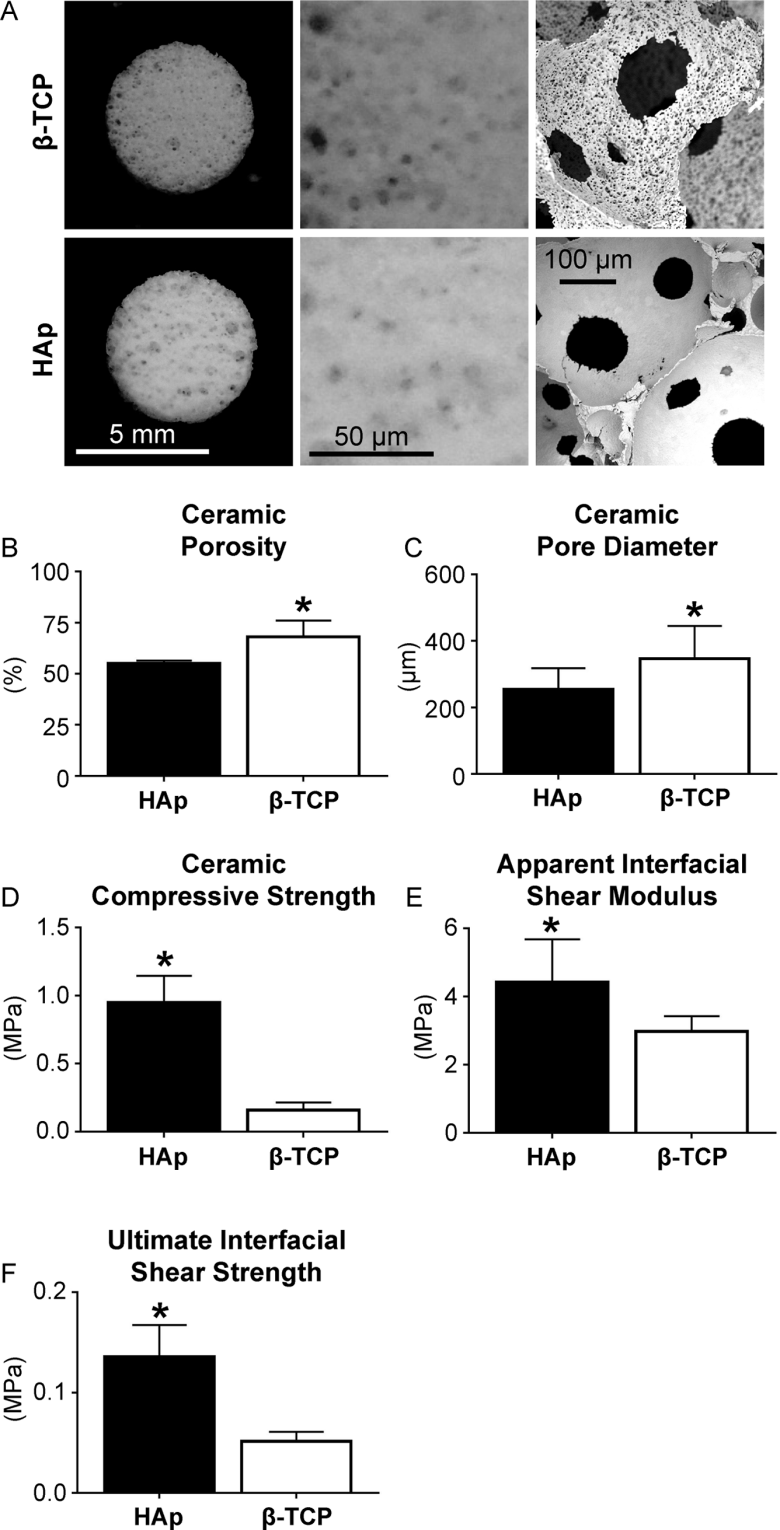
Characterization of HAp and β-TCP ceramics. Pore microstructure is shown (A). Ceramic porosity and pore diameter of the β-TCP ceramics exceeded those of the HAp ceramics (B, C). However, the compressive strength of the HAp ceramics exceeded that of the β-TCP ceramics (D) and the osteochondral interfacial shear modulus and strength of the osteochondral constructs composed of HAp exceeded those composed of β-TCP (E, F).

The ceramic porosity, pore diameter, compressive strength, and osteochondral interfacial properties were characterized (Fig 2). It should be noted that during interfacial testing of the constructs, failure of HAp osteochondral constructs consistently occurred at the neocartilage-ceramic interface. In contrast, failure of β-TCP osteochondral constructs consistently occurred within the ceramics, not at the neocartilage-ceramic interface. Both ceramics satisfied the porosity, pore diameter, and compressive strength requirements. However, due to the weakness of the β-TCP ceramic during interfacial shear testing, as well as its low compressive strength, the HAp ceramic was carried forward to Study 2.

### Construct gross morphology and histology

**Fig 3:**
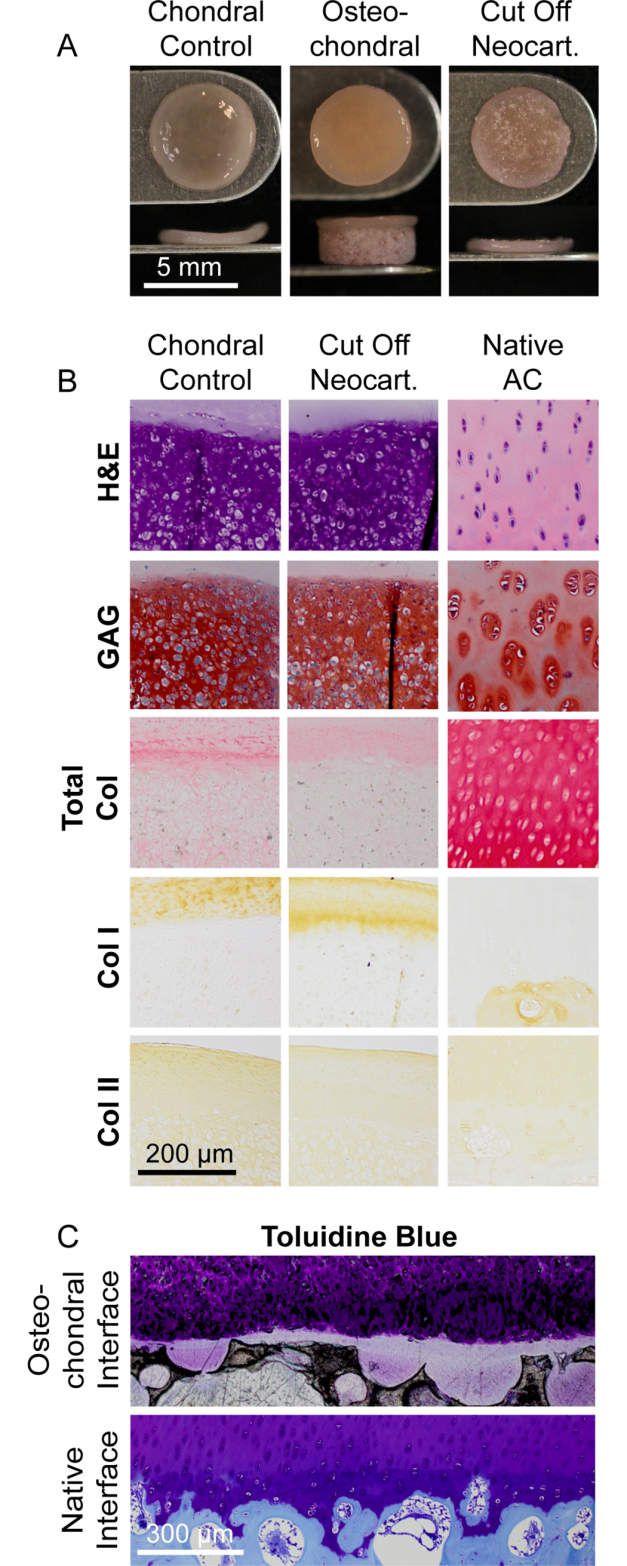
Gross morphology and histology of chondral control neocartilage, osteochondral, and cut off neocartilage. Construct gross morphology (A), chondral control and cut off neocartilage histology (B), and osteochondral histology (C) are shown.

Grossly, the neocartilage grown as the chondral control and the neocartilage grown as part of the osteochondral construct did not appear different (Fig 3). However, the neocartilage thickness of the chondral control group was significantly greater than that of the neocartilage cut off the osteochondral constructs, 0.52 ± 0.04 and 0.38 ± 0.03 mm, respectively. Histologically, the interior of both the chondral control and cut off neocartilage was cellularly dense and rich in GAG and collagen. The cells became less dense and smaller toward the periphery of the construct, with the chondrocytes at the edge becoming elongated parallel to the surface. Around the cellular area of the construct a collagen-rich outer layer was present. This outer layer was histologically visible on both sides of the chondral control group. The collagen-rich outer layer on the cut off neocartilage was only histologically present on the non-interfacial side because when the osteochondral constructs were dissected, the bottom collagenous layer remained within the pores of the ceramic. The chondral control and cut off neocartilage stained similarly for GAG, collagen I, and collagen II. Collagen I staining for both groups was localized to the outer layer, whereas collagen II stained throughout the depth of the tissues. Total collagen staining was less intense in the center but more intense in the outer layer of the cut off neocartilage group. Toluidine blue staining of the osteochondral interface showed the outer, collagen-rich layer of neocartilage interdigitated within the pores of the HAp ceramic.

### Neocartilage biochemical content

**Fig 4:**
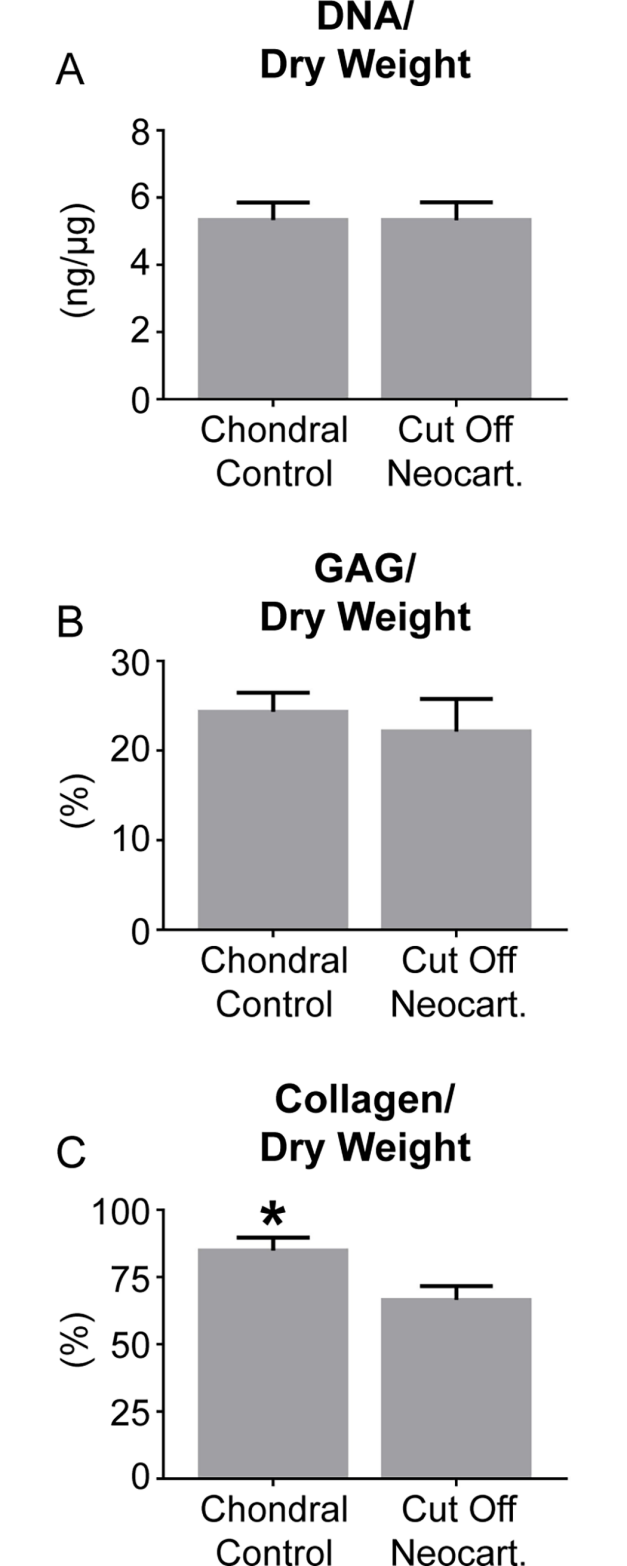
Biochemical compositions of chondral control and cut off neocartilage. DNA/dry weight (A), GAG/dry weight (B), and collagen/dry weight (C) contents of chondral control and cut off neocartilage constructs are shown.

The biochemical content normalized to dry weight of the chondral control and cut off neocartilage is shown in Fig 4. The water content of the chondral control neocartilage was significantly greater than that of the cut off control neocartilage: 86.7 ± 1.9 and 84.0 ± 2.5% respectively. The DNA/wet weight of the cut off neocartilage was significantly greater than that of the chondral control neocartilage: 0.8 ± 0.1 and 0.7 ± 0.1 ng/μg, respectively. The GAG/wet weight of the chondral control and cut off neocartilage was 3.2 ± 0.5 and 3.5 ± 0.6%, respectively. The GAG/DNA of the chondral control and cut off neocartilage was 0.2 ± 0.0 and 0.2 ± 0.0 μg/μg, respectively. The collagen/wet weight of the chondral control and cut off neocartilage was 2.3 ± 0.4 and 2.1 ± 0.4%, respectively. The collagen/DNA of the chondral control and cut off neocartilage was 2.4 ± 0.5 and 2.3 ± 0.4 μg/μg, respectively.

### Construct mechanical properties

**Fig 5:**
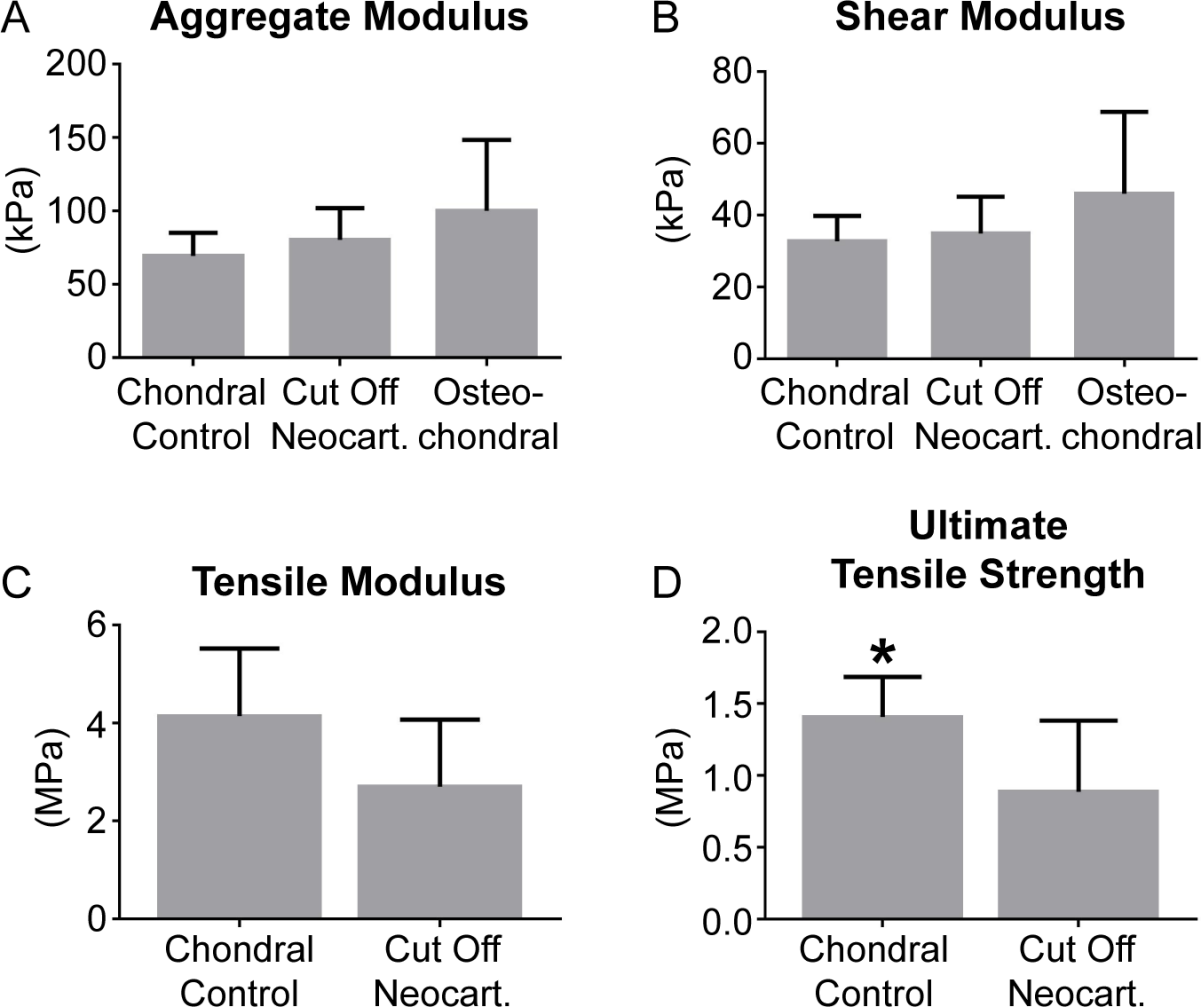
Mechanical properties of chondral control neocartilage, osteochondral, and cut off neocartilage constructs. The aggregate moduli (A), shear moduli (B), tensile moduli (C), and ultimate tensile strengths (D) of the chondral control neocartilage, osteochondral, and cut off neocartilage constructs are shown.

Results of creep indentation compressive testing and uniaxial tensile testing on the chondral control, cut off neocartilage, and osteochondral constructs are shown in Fig 5. The permeability of the chondral control neocartilage, cut off neocartilage, and osteochondral constructs was 2.9 ± 2.1, 7.2 ± 5.3, and 9.9 ± 7.7 x10^15^ m^4^/Ns, respectively. All samples failed within the gauge length during uniaxial tension testing.

## Discussion

The overall objective of this work was to engineer an osteochondral construct with a chondral phase composed of robust, self-assembled neocartilage interdigitated with a ceramic osseous phase that would be amenable to bone ingrowth. In the first study, the properties of HAp and β-TCP ceramics formed using a foam casting method were examined. While both ceramics were amenable to neocartilage interdigitation, the HAp ceramic was selected to carry forward to the second study because of its superior compressive properties (6 times stronger than β-TCP). In the second study, using the HAp ceramic composition, it was shown that incorporation of the ceramic in neocartilage culture did not interfere with the self-assembling process. The functional properties of neocartilage cut off the osteochondral constructs were found to be on par with chondral control neocartilage. Only collagen/dry weight and ultimate tensile strength of the chondral control remained significantly greater than the neocartilage cut off the osteochondral constructs, likely due to the physical act of dissecting the neocartilage off the ceramic in the osteochondral constructs. This series of studies shows that it is possible to engineer an osteochondral construct with a robust chondral phase and makes strides toward developing methods with which to do so.

This study demonstrated the feasibility of engineering an osteochondral construct with a robust interface. Ceramics, such as HAp and β-TCP are widely used as bone substitutes in orthopedic surgery [15, 16]. The use of these as the osseous phase of an osteochondral construct is advantageous, not only because of their non-toxicity and biocompatibility, but also because the porosity and resorption may be tailored to match the rate of new bone formation *in vivo*. Great strides have been made toward engineering biomimetic neocartilage *in vitro*. We have previously reported native-like structure [18], biochemical composition [19], and mechanical properties [20] of scaffold-free, self-assembled engineered neocartilage. The use of self-assembled neocartilage as the chondral phase of an osteochondral construct is beneficial because its composition, structure, and mechanics allow for immediate loading upon implantation and it does not contain a scaffold which may obstruct construct integration or remodeling *in vivo*. This study successfully combined these osseous and chondral phases in a repeatable manner which yielded consistent and robust mechanics of the osteochondral interface and construct as a whole. For the HAp osteochondral construct, the apparent shear modulus of the osteochondral interface was 4.4 MPa (Fig 2). Furthermore, neocartilage interdigitation into the pores of the HAp ceramic was seen microscopically (Fig 3). The repeatable osteochondral assembly method may serve as a template that allows for the flexibility to utilize ceramics of varying compositions and scaffold-free engineered neocartilage composed of different cell types under various stimulation regimens. The osteochondral constructs resulting from this method make strides toward engineering mechanically robust, biomimetic grafts which integrate into and, ultimately, regenerate cartilage defects.

Because robust functional properties of neocartilage are achieved via the self-assembling process, it was crucial to examine the effect of incorporating a ceramic in neocartilage culture. The self-assembling process does not utilize scaffolds or external forces to engineer neocartilage [25]. The process takes place in a non-adherent environment and is based on cell-to-cell binding via free energy minimization. Study 1 importantly shows that the inclusion of an HAp ceramic, which is essentially a scaffold for native bone integration, in self-assembly culture does not alter chondrocyte phenotype, disrupt neocartilage formation, or alter neocartilage functional properties (Figs 3–5). The self-assembling process occurs in four phases characterized by 1) seeding of a high density of cells in a non-adherent environment, 2) integrin and N-cadherin mediated cell-to-cell interactions and tissue formation [26], 3) production of immature cartilage ECM, and 4) ECM maturation [25]. Osteochondral assembly occurred at day 10, likely during the transition between phase 3 and 4 of the self-assembling process. This timing was sufficiently late in the process to not affect the cellular interactions or tissue formation processes crucial to making robust neocartilage. Incorporation of an HAp ceramic in neocartilage culture did not disrupt the self-assembling process and did not alter the resulting neocartilage functional properties.

In this study, the HAp and β-TCP ceramics composing the osseous phase of an osteochondral implant were formed using a novel foam casting method. Many osseous phase scaffolds are composed of non-ceramic materials, or contain non-ceramic materials in addition to HAp, β-TCP, or calcium polyphosphate (CCP). Silk [27, 28], polylactic acid (PLA) [1], poly(lactic-co-glycolic) acid (PLGA), polycaprolactone [29], and ECM proteins are frequently used. Gravity sintering [30–33] and the formation of an aqueous paste or gel which is then freeze-dried and sintered [34–36] are commonly used techniques to form pure ceramic scaffolds of various compositions. With these techniques, the ceramics are typically molded into the final shape before sintering, or further modified by cutting after sintering. The novel ceramic engineering method used in this study involves the formation of an aqueous foam containing the HAp or β-TCP microparticles, polyethyleneimine, and glycerol diglycidyl ether. This mixture also contains a surfactant to create manipulatable porosity. The foam is molded into blocks which polymerize via an epoxide reaction. After polymerization, the blocks are demolded, frozen, and lyophilized to stabilize them, which allows for room temperature storage. Stabilized blocks may then be machined into posts, as in this study, or to any custom shape, which has great clinical importance. After machining, the ceramics are sintered. Sintered ceramics are brittle, so machining the final shape prior to sintering allows for more complex geometries to be achieved. While a similar ceramic formation process has been reported [37], the unique and significant aspects of the methods developed in this study (the use of a surfactant, the room temperature stability of ceramic blocks prior to sintering, and the flexibility of the final architecture of the ceramic) allow for “off the shelf” product development, customization of porosity by manipulation of surfactant volumes, and customization of ceramic size and geometries to suit specific purposes or individual patients. The method developed in this study was tailored to the formation of a trabecular-like bone substitute, and its suitability for other purposes should be explored in future studies. The ceramic formation method developed in this study has the potential for the development of “off the shelf” ceramics and ceramics with customizable characteristics such as porosity and overall size and shape, both of which may be advantageous strategies in clinical use.

While the ceramics composed of β-TCP were significantly weaker than those composed of HAp, the inclusion of this material should not be ruled out. Ceramic engineering criteria to allow for bone ingrowth in potential *in vivo* studies were identified as a pore diameter of 150–500 μm [12], a porosity of 50–90% [13], and a minimum compressive strength of 0.1 MPa [13, 14]. Both the HAp and β-TCP ceramics showed acceptable physical characteristics as they both satisfied all these criteria (Fig 2). However, during the lap shear evaluation of the ability of each ceramic composition to allow for neocartilage interdigitation (Study 1), the β-TCP osteochondral constructs consistently failed within the ceramic, not at the neocartilage-ceramic interface. This unintended, but nevertheless important, finding suggests that in addition to β-TCP ceramics being significantly weaker than HAp ceramics in compression, they are also weaker in tension and shear. The weakness of the β-TCP ceramics may be attributed to the significantly greater porosity and macropore size, as well as the presence of micropores, compared to the HAp ceramics. While the use of pure β-TCP may not be mechanically suitable for use as the osseous phase of osteochondral constructs, it has promising osteoconductive characteristics. β-TCP has higher solubility than HAp, is degradable via osteoclastic activity, and is used commercially as Conduit TCP Granules and chronOS strips (DePuy Orthopaedics) [17, 38]. Ceramics containing both β-TCP and HAp, termed biphasic calcium phosphates (BCPs), are desirable for bone regeneration because their degradation characteristics are customizable by altering the relative amounts of β-TCP and HAp [17]. The inclusion of β-TCP may not only be desirable, but also essential to allow for immediate bone turnover [39]. It has also been suggested that the use of BCPs allows osteoclasts to act in a more natural way than pure HAp or β-TCP [39]. The desirable characteristics of both HAp and β-TCP motivate the future study of ceramics containing mixed compositions for use in osteochondral constructs.

Within neocartilage of both groups, the preliminary organization of zones may be present. Within the interior of both the chondral control and cut off neocartilage, chondrocytes were densely packed in a GAG and collagen-rich ECM. Toward the periphery of the constructs, chondrocytes became smaller and less densely arranged, GAG staining became less intense, and collagen staining became more intense. At the periphery of the construct, chondrocytes appeared small and elongated parallel to the edge of the cellular region. Beyond the cellular region was as mostly acellular, collagen-rich layer. These patterns parallel the zonal organization of native tissue [7]. In the middle zone of articular cartilage, proteoglycan content is high and collagen content is low. Toward the superficial zone, proteoglycan content decreases and collagen content increases. Zonal organization has been observed previously within self-assembled caprine articular chondrocytes [40]. During dissection of the osteochondral constructs for testing, a portion of the collagen-rich outer layer of the cut off neocartilage remained within the pores of the ceramic. This may be responsible for not only the lower collagen/dry weight content in the cut off neocartilage group, but also its lower tensile strength. It has been previously shown that removal of the collagen-rich, superficial zone of native cartilage results in decreased tensile properties [41]. Additionally, the physical act of removing the neocartilage that had interdigitated with the ceramic may have introduced defects into the tissue which resulted in stress concentrations and modified tensile properties. The neocartilage grown as the chondral phase of an osteochondral construct was not functionally different than chondral control neocartilage, with both groups showing zonal organization reminiscent of native cartilage.

## Conclusions

This study makes strides toward creating a biphasic osteochondral construct composed of robust, scaffold-free neocartilage and osteoconductive ceramic. It also provides the foundation for creating “off the shelf” and highly customizable bone analogs using a novel ceramic formation method. The data presented here also show that the inclusion of an HAp ceramic does not impact the self-assembling process and results in the interdigitation into the ceramic of functionally robust neocartilage with zonal organization. Future studies should explore the possibility of engineering larger ceramics of complex shapes, perhaps with a combination of HAp and β-TCP. The interdigitation of the two phases and interfacial mechanical properties may also be improved. Finally, animal studies should be performed to examine the cartilage healing response and integration of these osteochondral constructs *in vivo*.
